# 14-3-3ε Is Required for Germ Cell Migration in *Drosophila*

**DOI:** 10.1371/journal.pone.0036702

**Published:** 2012-05-30

**Authors:** K. Kirki Tsigkari, Summer F. Acevedo, Efthimios M. C. Skoulakis

**Affiliations:** Biomedical Sciences Research Centre “Alexander Fleming”, Vari, Greece; Stockholm University, Sweden

## Abstract

Although 14-3-3 proteins participate in multiple biological processes, isoform-specific specialized functions, as well as functional redundancy are emerging with tissue and developmental stage-specificity. Accordingly, the two 14-3-3ε proteins in Drosophila exhibit functional specificity and redundancy. Homozygotes for loss of function alleles of *D14-3-3ε* contain significantly fewer germ line cells (pole cells) in their gonads, a phenotype not shared by mutants in the other 14-3-3 gene *leo*. We show that although D14-3-3ε is enriched within pole cells it is required in mesodermal somatic gonad precursor cells which guide pole cells in their migration through the mesoderm and coalesce with them to form the embryonic gonad. Loss of D14-3-3ε results in defective pole cell migration, reduced pole cell number. We present evidence that D14-3-3ε loss results in reduction or loss of the transcription factor Zfh-1, one of the main regulatory molecules of the pole cell migration, from the somatic gonad precursor cells.

## Introduction

The 14-3-3 proteins are small dimeric acidic proteins which are highly conserved throughout the eukaryotes [1], [2], [3]. Multiple family members exist in metazoans, characterized by exceptionally high sequence similarity among homologous isotypes from different species. On the basis sequence identity the 9 mammalian species form two evolutionary conservation groups [4].. Although these proteins are highly abundant in the nervous system, they are also present in an apparently isotype-specific manner in many other tissues including multiple organ and glands, the retina, the ovaries and testes in vertebrates and invertebrates [1], [2], [5].

14-3-3 proteins interact with diverse cellular proteins mostly by binding to phosphorylated Serines or Threonines within particular motifs and have been associated with many cellular processes and functions [6], [7]. 14-3-3 binding to clients may enhance or suppress their interactions with other proteins, which could prevent or enhance post-translational modifications or stability of the target proteins. In addition, interactions with 14-3-3 proteins are known to regulate catalytic activity or sub-cellular localization of client proteins [8]. In some cases, 14-3-3s engage more than one of these mechanisms to regulate the function of their targets [9]. For such effects to be biologically relevant the affinity and specificity of interactions with clients are likely to be isotype-specific and in fact *in vivo* specificity for 14-3-3 isotype-client interactions has been reported. For example, Cdc25C interacts with the 14-3-3ζ, ε and γ isotypes and not with the 14-3-3σ isotype [10], whereas Cdc25B protein interacts preferentially with the 14-3-3β, η, and ζ isotypes [11].

Drosophila contains only two 14-3-3 genes, *leonardo*, an ortholog of the mammalian *14-3-3ζ* and *D14-3-3ε* [4]. The *leonardo* gene encodes three nearly identical protein isoforms (LeoI, LeoII and LeoIII) through alternative splicing of the primary transcript [12]. Of these, LeoIII appears to be the most spatially restricted to adult mushroom body neurons and LeoI the most ubiquitous [12]. In contrast, *D14-3-3ε* encodes a single protein [13], [14], present in all developmental stages and tissues examined [13], [15]. Because Leo and D14-3-3ε represent the two different conservation groups, Drosophila offers a simple but representative system to investigate 14-3-3 functions and specificity *in vivo*. In fact, these two fly 14-3-3 proteins have been reported redundant in particular biological functions in a tissue-specific manner, but not for others [13]. Maternally originating Leo and D14-3-3ε appear to have distinct roles in chromosome segregation and the synchronization of syncytial mitoses [16]. D14-3-3ε plays a role in photoreceptor specification in the developing eye, where it can be functionally complemented by Leo isoforms [14], [17]. In contrast, the requirement for D14-3-3ε in wing cross-vein formation cannot be functionally complemented by Leo [13]. Moreover, LeoI and to a lesser extend LeoII can functionally compensate for D14-3-3ε loss in processes underlying vital functions essential for late embryonic development [13].

An additional phenotype which characterizes *D14-3-3ε* null mutant homozygotes is sterility [14] and we aimed to determine the cause of this novel phenotype. In addition, in the context of our work on Drosophila 14-3-3 functional specificity, we wondered whether the deficit can be functionally complemented by Leo. In this study, we demonstrate that D14-3-3ε regulates the stability of Zinc finger homeodomain protein-1 (Zfh-1), a transcription factor essential for formation and function of the mesodermally-derived somatic part of the embryonic gonad.

Cellular movements play a crucial role in the development of multicellular organisms and can serve a variety of functions ranging from generation of different tissue layers during gastrulation to organogenesis. These cellular migrations bring into contact different cell types, which is often required for their final differentiation. The migration of primordial germ cells (PGCs) provides a model to study cellular movement and differentiation during development [18], [19]. In many organisms including the Drosophila embryo, germ cells form in a position distinct from the final location of the gonad. Fly PGCs often referred to as pole cells, are the first to cellularize at the posterior pole of the embryo (stage 5). At gastrulation they move along the dorsal surface of the embryo and are incorporated into the invaginating posterior midgut (PMG) pocket (stage 8). Then, the PGCs migrate through the PMG wall, moving along its basal surface to the dorsal side of the embryo (stage 9). From this position, they move toward and eventually align with mesodermal cells that will give rise to the somatic component of the gonad (stages 12-13). Finally, the PGCs and gonadal mesoderm coalesce to form the embryonic gonad (stage 14). Consequently, germ cell migration in Drosophila provides a model system for the study of cell-cell interactions and cellular movements through and along different tissue layers [20], [21]. A number of gene products necessary for pole cell migration and eventual interaction with the somatic component of the gonad have been identified [22] and the work described herein demonstrates that D14-3-3ε is an additional member of the group.

## Results

### D14-3-3ε is required for pole cell migration to the embryonic gonads

**Figure 1:**
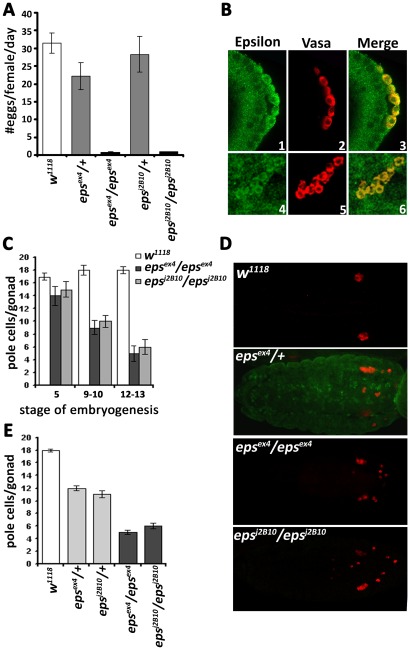
Reduction in the pole cell number in *D14-3-3ε* mutant embryos. A. Reduced fecundity of homozygous mutant females reflected in the number of eggs laid per single female per day. Homozygous *D14-3-3ε^ex4^* and *D14-3-3ε^J2B10^* lay very few eggs (1 or 2 per day), while *D14-3-3ε^ex4^* heterozygotes also exhibit significantly reduced fecundity compared to controls. B. Wild type embryos of stage 5 (1–3) and stage 11–12 (4–6) stained with anti-ε (green) and a-vasa (red). D14-3-3ε is expressed inside and outside of the pole cells. C. Quantification of the number of pole cells that reach each embryonic gonad estimated from at least 12 different embryos. The numberof pole cells per gonad of heterozygous (light gray bars) and homozygous (dark gray bars) mutant embryos is significantly different (p<0.001, Dunnett's test) from that in controls (open bars). D. Pole cell distribution in 16–18 hr homozygous mutants and *D14-3-3ε^ex4^*/TM3GFP heterozygotes. anti-GFP staining (green) was used to distinguish heterozygotes from homozygous mutant embryos. Unlike in similarly aged control embryos, pole cells in heterozygous and homozygous mutant embryos appear dispersed all over the embryo and result in the formation of gonads with fewer pole cells. C. Quantification of the total number of pole cells per gonad in control and homozygous mutant embryos (n≥12 each) carefully staged according to morphological criteria. Although consistently somewhat reduced compared to controls at stage 5, pole cell number in mutant embryos became highly significantly different (p<0.001, Dunnett's test) from that in controls at stage 8 and 11–12.

Male and female *D14-3-3ε* null mutants homozygous for the deletion *D14-3-3ε^ex4^* or the transposon insertion *D14-3-3ε^l(3)j2B10^* were reported sterile [13], [14]. Our own results verified these reports and demonstrated that the sterility did not have behavioral origins as all male and female mutant homozygotes were observed to mate with the respective tester *w^1118^* animals (Table 1). This analysis also revealed that *w^1118^* tester females after mating with *D14-3-3ε* null males laid ample, but apparently infertile eggs (Table 1). In contrast, *D14-3-3ε* null females mated with *w^1118^* tester males laid very few also infertile eggs. Quantification of the fecundity deficit demonstrated that whereas control females yielded approximately 30 eggs, mutant homozygote females laid only 1-2 daily (Fig. 1A). Even *D14-3-3ε^ex4^* (but not *D14-3-3ε^l(3)j2B10^/+*), heterozygous females laid significantly less eggs (∼18) than controls. The phenotype is a consequence of the mutations in *D14-3-3ε* as it is readily rescued by trangenes carrying full length *D14-3-3ε* cDNA under the ubiquitously expressed heat-shock promoter induced twice daily throughout development (Table S1). Therefore, we hypothesized that the apparent rarefaction of eggs and sperm upon D14-3-3ε loss may reflect defective adult gametogenesis, or defective gonadal development, or both.

**Table 1:** *D14-3-3ε* mutants are sterile.

Genotype	Female		Male	
	# Crossed	% Fertile	# Crossed	% Fertile
*D14-3-3ε ^ex5^/ D14-3-3ε ^ex5^*	20	100	20	100
*D14-3-3ε ^l(3)j2B10^/ D14-3-3ε ^ex5^*	20	100	20	100
*D14-3-3ε ^l(3)j2B10^/ D14-3-3ε ^l(3)j2B10^*	30	0	27	0
*D14-3-3ε ^l(3)j2B10^/ D14-3-3ε ^ex4^*	30	0	25	0
*D14-3-3ε ^l(3)j2B10^/ D14-3-3ε ^ex24^*	23	0	21	0
*D14-3-3ε ^ex4^/ D14-3-3ε ^ex5^*	18	100	18	100
*D14-3-3ε ^ex4^/ D14-3-3ε ^ex4^*	25	0	26	0
*D14-3-3ε ^ex4^/ D14-3-3ε ^ex24^*	21	0	18	0
*w^1118^*	25	100	25	100

The number of single crosses that yielded larvae (% Fertile) over the total number of animals crossed (# crossed) per genotype is reported. D14*-3-3ε^ex5^* are used as controls since they have the same genetic background as the mutants and express normal amounts of the protein [13].

Although rather ubiquitous [13], D14-3-3ε is also present inside pole cells and the surrounding tissues from early stage 5 (Fig. 1B) to the end of embryonic development. Hence, we focused on the possibility of defective gonadal development in homozygous mutant embryos as potentially causal of the fertility and fecundity defects. Homozygous and heterozygous mutants were identified on the basis of GFP fluorescence conferred by the balancer chromosome (see Materials and Methods). Although somewhat fewer, stage 5 homozygous mutant embryos did not contain significantly less pole cells (Fig.1C) to explain the fertility and fecundity defect. However, in these mutant embryos, pole cells failed to coalesce in the embryonic gonad at later stages and appeared scattered throughout the caudal side. Only few reached the endpoint of their migration through the mesoderm and appeared to take residence in the embryonic gonad (Fig. 1D). A similar, albeit milder distribution of pole cells outside the gonad at the end of embryogenesis was observed in *D14-3-3ε^ex4^* heterozygotes (Fig. 1D).

Quantification demonstrated three distinct statistically different distributions of pole cell numbers coincident with the level of D14-3-3ε. Control gonads contained 18–20 pole cells each, those of heterozygous mutant embryos 10–12, while only approximately 7 pole cells were found in the gonads of mutant homozygotes (Fig.1E). The distribution of pole cells outside the gonad in heterozygous and homozygous mutant embryos, suggests impaired migration through the mesoderm [19]. In fact, quantification of the total number of pole cells in different stages of embryogenesis revealed that in stage 5 embryos, the number of pole cells in mutant homozygotes is similar with that in controls. However, whereas pole cell number remained relatively constant thereafter in control embryos, there was a large reduction in their number by stage 8 in the mutants, which apparently continued to decline yielding 4–6 pole cells reaching each gonad by stage 11–13 (Fig. 1C). These results are consistent with the notion that pole cell number reduction in mutant homozygotes is due to death of cells failing to reach and coalesce in the gonad. Collectively, the data indicate that D14-3-3ε function is required for efficient pole cell migration to the embryonic gonad and that abortive pole cell migration in *D14-3-*3ε mutants and the resultant reduction in germ cells could be at least a partial explanation for their sterility.

### D14-3-3ε is required in mesoderm for normal pole cell migration

**Figure 2:**
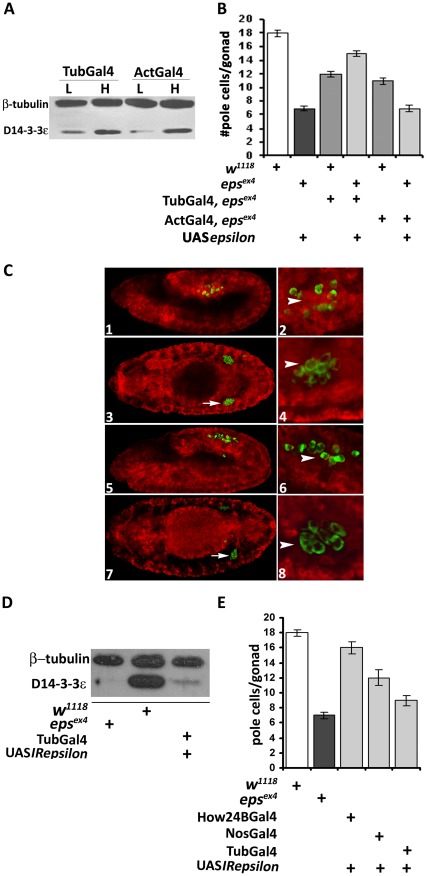
Cell type limited transgenic rescue and phenocopy of the pole cell deficit. A. Equivalent expression of two independent *UASepsilon* transgenes in different chromosomal locations expressing either low (L) or high (H) levels of transgenic protein under either Tub-Gal4 or Act-Gal4. B. Transgenic rescue of the scattered pole cell phenotype of *D14-3-3ε^ex4^* homozygous and heterozygous embryos under the Tub-Gal4 or Act-Gal4 drivers. The dark bar indicates the number of pole cells per gonad in *D14-3-3ε^ex4^* homozygotes carrying a silent (no Gal4 driver) *UASepsilon* transgene. Pole cell number was significantly (p<0.0001, Dunnett's test) higher in mutant heterozygotes (medium gray bars) than in *D14-3-3ε^ex4^* homozygotes. Importantly, expression of the transgene under Tub-Gal4 (middle light gray bar) resulted in a significant increase (p<0.0001, Dunnett's test) of pole cells per gonad over those in mutant homozygotes (dark bar). In contrast, pole cell number remained similar to that of the homozygous mutants under Act-Gal4 (rightmost light gray bar). C. Expression pattern of Tub-Gal4 and Act-Gal4 drivers. Expression of the transgenic protein b-Tau (red) using the drivers Tub-Gal4 (1–4) and Act-Gal4 (5–8) and in the embryonic stages 11 (1, 2, 5 and 6) and 14 (5, 6, 7 and 8). Pole cells are labeled with anti-vasa (green) and the arrows indicate their location in late with respect to cells expressing the Tub-Gal4 (3) and Act-Gal4 (7). Arrowheads in 2 and 4 indicate Tub-Gal4 driver expression in mesodermal cells surrounding the migrating pole cells and the of transgenic protein expression under Act-Gal4 in these cells (6, 8). D. RNAi-mediated attenuation of endogenous D14-3-3ε protein upon ubiquitous expression under Tub-Gal4 in embryos. The western blot probed with the chicken anti-D14-3-3ε antibody is also probed with anti-β-tubulin which serves as a loading control and demonstrates drastic reduction of D14-3-3ε to levels nearly as low as those in homozygous mutant embryos. E. RNA-interference(RNAi)-mediated phenocopy in wild type embryos of the pole cell deficit in *D14-3-3ε^ex4^*homozygotes. Driving the RNAi-mediated transgene with Tub-Gal4 reduced pole cell number in the gonads of stage 12–13 embryos nearly to that observed in mutant homozygotes (dark gray bar). Partial, but statistically significant (p<0.001, Dunnett's test) reduction compared to controls (open bar) was attained under NosVp16-Gal4, but no deficit was precipitated under the mesodermal driver How24B-Gal4.

Transgenic rescue of the mutant phenotype was attempted to unequivocally demonstrate that the deficit in pole cell migration was consequent of tissue-specific D14-3-3ε loss. Secondly, we wondered whether the deficit originates from loss of the protein within the pole cells consistent with the presence of the protein there, or elsewhere within the embryo. We initially attempted rescue under the ubiquitous drivers Tub-Gal4 and Act-Gal4 to emulate the wide distribution of D14-3-3ε in embryos [13]. Two independent UAS driven *D14-3-*3ε transgenes were utilized, which due to apparent position effects yield high (H) and low (L) transgenic protein under both drivers (Fig. 2A). Therefore, the two drivers do not differ significantly with respect to the levels of transgenic protein they induce. However, whereas near full rescue of pole cell number was achieved with the high expressing transgene under Tub-Gal4, it completely failed under Act-Gal4 (Fig. 2B). Because this difference could not be attributed to different overall levels of transgenic protein under these two drivers, we investigated potential differences in their spatial expression. We made the assumption that such putative spatial expression differences, however small, might account for the rather large differences in phenotypic rescue. To increase the resolution of immunohistochemical detection of potential spatial expression differences, we drove a transgene expressing the bovine Tau protein (bTau), which as previously shown [23], yields a highly defined and specific signal. As demonstrated in Fig. 2C neither driver is expressed within the pole cells. Importantly, Tub-Gal4 drives reporter expression in cells surrounding and apparently in direct contact with pole cells in stage 11 and stage 15 embryos (Fig. 2C1–2C4). These mesodermal cells are known to play important roles in the guidance and survival of pole cells throughout their migration to the forming gonad [24], [25]. These Somatic Gonadal Precursor (SGP) cells form in parasegments 10–12 in the caudal area and germ cells migrate towards them. By stage 13 the two cell types coalesce into the gonad which contains both germ and somatic cells [19]. In contrast, Act-Gal4 is not expressed in mesodermal tissues proximal to pole cells (Fig. 2C5–2C8) and this may underlie the lack of rescue under this driver. Therefore, the driver expression pattern combined with the results of the rescue experiments indicate that direct contact between mesodermal and pole cells is required for full rescue and importantly, D14-3-3ε appears to be required in the mesoderm for this interaction.

To verify these conclusions independently, we generated a transgene capable of producing an interfering RNA (RNAi), and obtained multiple lines capable of abrogating the endogenous D14-3-3ε (Fig. S1). We used the insertion on the X due to its highest efficacy (Fig S1B), but results were also verified with an additional independent RNAi transgenic line. In fact, ubiquitous expression of the RNAi-mediating transgene nearly abolished D14-3-3ε levels in late embryos (Fig. 2D), with the perduring protein potentially being of maternal origin [13]. Abrogation of D14-3-3ε under Tub-Gal4 phenocopied pole cell loss in otherwise wild type embryos (Fig. 2E), while abrogation of the protein in mesodermal cells under How24B-Gal4, did not result in significant reduction of pole cell number. However under the caudal mesoderm driver NosVp16-Gal4, which is also expressed in pole cells, there was a small but significant reduction (Fig. 2E). This is consistent with the requirement for D14-3-3ε in the caudal mesoderm and likely also reflects differences in the spatial expression patterns of the two drivers. However, as experiments under NosVp16-Gal4 did not yield embryonic gonads with the normal number of pole cells (not shown), it is probable that expression levels under this driver remain below the required threshold for full phenocopy or rescue of the pole cell deficit. Nevertheless, these results verify the outcome of the rescue experiments (Fig. 2B) and indicate that D14-3-3ε is necessary in posterior mesoderm cells probably the SGPs and subsequent in somatic gonadal cells, where How24B is apparently not expressed.

### Specificity in 14-3-3 protein requirement for pole cell migration

**Figure 3:**
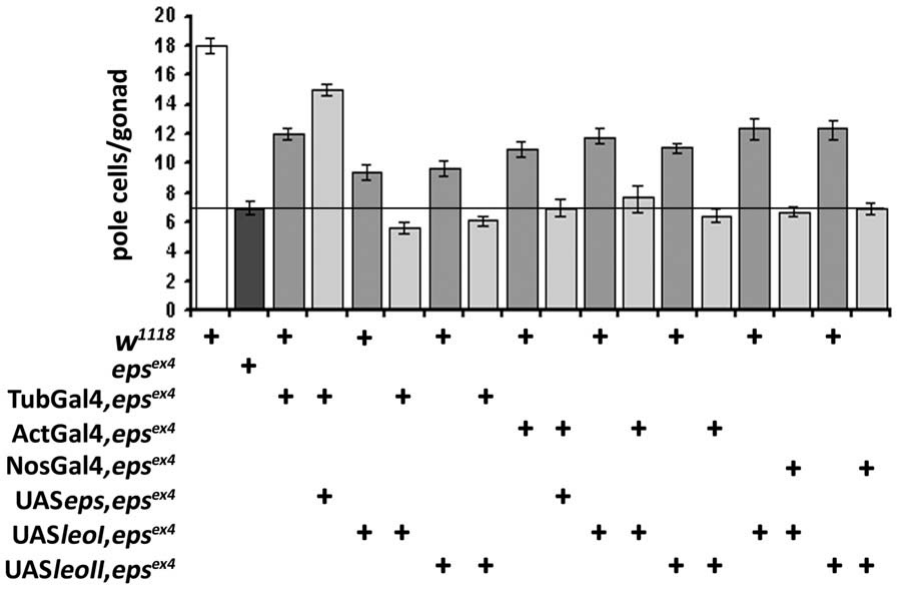
14-3-3ζ-Leo over-expression does not rescue pole cell number in *D14-3-3ε^ex4^*homozygotes. Expression of the two ubiquitous Leo isoforms, LeoI and LeoII [12] in mutant homozygotes under Tub-Gal4, Act-Gal4 and NosVp16-Gal4 (light gray bars) failed to change the reduced number of pole cells in *D14-3-3ε^ex4^* homozygotes. The dark bar indicates the number of pole cells per gonad in *D14-3-3ε^ex4^* homozygotes. The number of pole cells in embryos expressing transgenes (light gray bars) was compared to that of the homozygotes (dark gray bar) and of genotype-matched heterozygotes (medium gray bars) carrying each UAS transgene on the same chromosome as that bearing the *D14-3-3ε^ex4^* mutation. Full rescue was attained only when Tub-Gal4 drove the UAS*epsilon* (UAS*eps*) transgene. The line is drawn to aid comparisons with the number of pole cells in the mutant homozygotes.

Previous results have demonstrated functional redundancy between D14-3-3ε, LeoI and LeoII, for specific functions in particular tissues [13]. Therefore, we investigated whether the two 14-3-3 proteins may also be functionally redundant for pole cell migration and embryonic gonad formation. We approached this by attempting rescue of the deficient pole cell number in *D14-3-3ε* mutant embryos with transgenes encoding LeoI and LeoII, but did not include LeoIII in these experiments because it is specific to adult mushroom body neurons and is more divergent from the other two [12]. Quantification of pole cell number per gonad, demonstrated that none of the Leo isoforms was able to rescue the pole cell reduction in the gonad, or their abortive migration upon D14-3-3ε loss under the ubiquitous Tub-Gal4, Act-Gal4, or the caudal mesoderm driver NosVp16-Gal4 (Fig. 3). Therefore, the two Drosophila 14-3-3 proteins Leo and D14-3-3ε are not functionally equivalent with respect to the pole cell migration. In agreement with this conclusion, unlike for *D14-3-3ε* mutant heterozygotes (Fig. 1D, Fig. 2B, Fig. 3C), the number of pole cells in *leo* mutant heterozygotes is not different from that in wild type embryos. In addition, although *leo* mutant homozygotes die as late embryos with developmental defects [26], which hamper accurate estimation of their number, they do not appear to harbor significantly less pole cells (not shown).

### Interaction of D14-3-3ε with proteins essential for pole cell migration

Normal pole cell migration requires the protein products of several genes such as *serpent (srp)* and *huckebein (hkb)*, engaged in normal mesoderm development [27], [28], [29] and others that affect pole cell migration *per* se, such as wunen *(wun)*, necessary for pole cell migration along the basal surface of the midgut [30]. In addition, mutations in *Abdominal A (abdA)*, *Abdominal B (abdB)*, *tinman (tin)*, *heartless (htl), fear of intimacy (foi), trithorax (trx), trithoraxgleich (trg) και zinc-finger homeodomain-1 (zfh-1)* affect normal pole cell migration and result in defective gonad formation [22]. Interestingly, mutations in many of these genes precipitate changes highly reminiscent of the phenotype of *D14-3-3ε* homozygotes, namely scattered pole cells across the mesoderm and gonads devoid of, or containing few germ cells. A potential explanation for the phenotypic similarity in these loss-of-function mutants is that interaction among one or more of these proteins and D14-3-3ε is necessary for pole cell migration and/or gonad formation. In fact, an *in silico* search revealed that Abdominal A, Columbus, Trithorax and Zinc-finger homeodomain-1 proteins contain one or more potential 14-3-3 binding sites (Table S2). Hence, we investigated whether the distribution pattern of these proteins was altered in *D14-3-3ε* mutant embryos. We focused on Zfh-1 for two reasons. It is the only one of the group that contains a perfect match to the consensus of the most common 14-3-3 binding site, Arg-Ser-x-Ser-x-Pro (where a x is any aminoacid) and has a highly specific antibody [31] available.

### 14-3-3ε is required for Zfh-1 stability in the somatic gonadal cells

**Figure 4:**
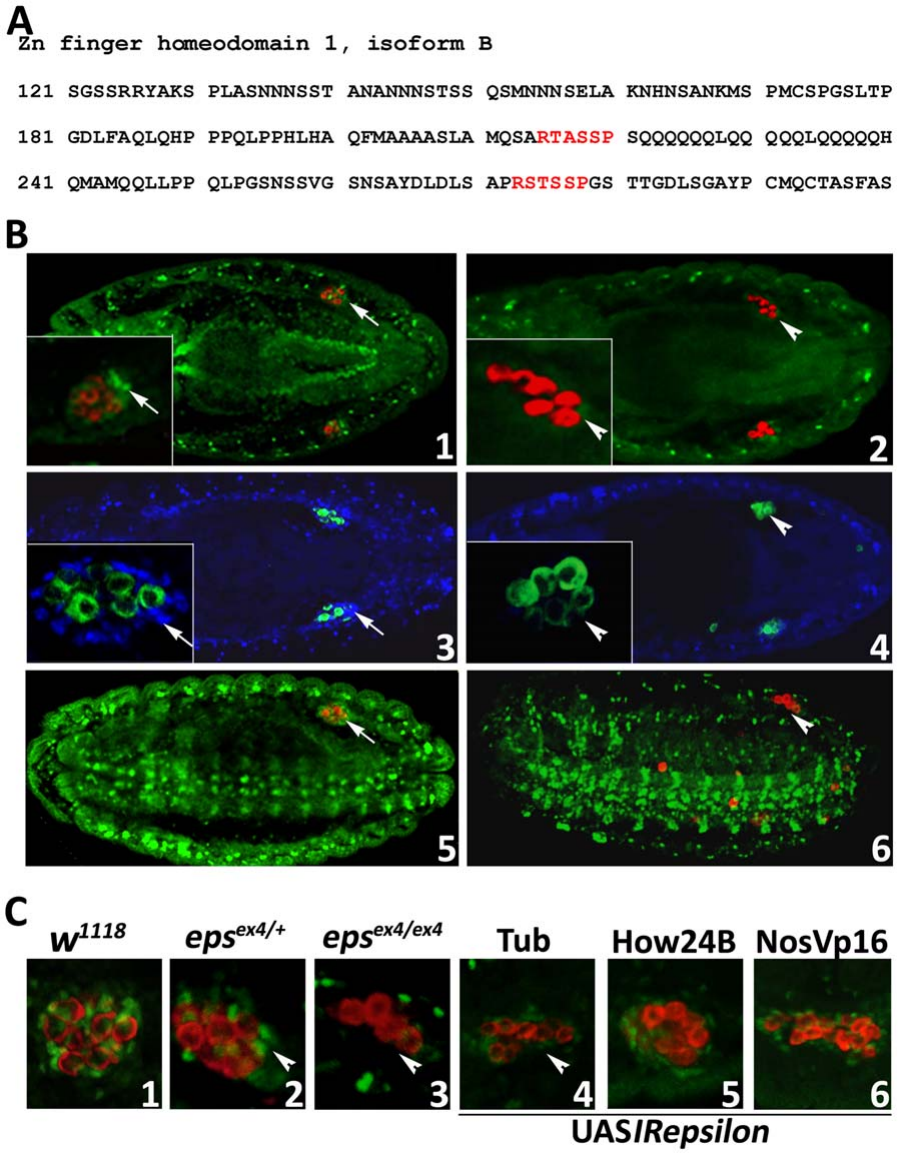
The Zfh-1 transcription factor interacts with and is regulated by D14-3-3ε. A. The amino acid sequence of Zfh-1 containing the two 14-3-3 binding sites (red) revealed by *in silico* analysis. The second site containing the amino acids RSTSSP represents a perfect fit to the typical consensus 14-3-3 binding. B. The Zfh-1 distribution in control (1, 5), *D14-3-3ε^ex4^* (2) and *D14-3-3ε^J2B10^* (6) homozygous mutant embryos is shown in green, while pole cells are stained for Vasa (red). In the independent experiment shown in panel 3 (control) and panel 4 (homozygous *D14-3-3ε^ex4^* mutant), the Zfh-1 distribution is shown in blue and pole cells marked with anti-Vasa in green. The inserts are magnifications of one of the gonads to better reveal the severe reduction or absence (arrowheads in 2, 4, 6) of Zfh-1 in mesodermally derived gonadal cells (arrows in 1, 3, 5). In 5 and 6 the embryos are oriented and images were captured such as to reveal the distribution of Zfh-1 in the ventral nerve chord and other mesodermal tissues. Note the scattered pole cells in panel 6. C. Phenocopy of the reduction or loss (arrowheads) of Zfh-1 in mesodermally derived gonadal cells upon RNAi-mediated D14-3-3ε abrogation with Tub-Gal4 (4) and to a lesser degree with NosVp16-Gal4 (6) in comparison with the distribution of these cells in control (1), *D14-3-3ε^ex4^* heterozygous (2) and homozygous (3) mutant embryos.

Zfh-1 is a transcription factor with nine zinc fingers and a homeodomain. It is normally expressed in the central nervous system and in mesodermal tissues such as the dorsal vessel, the precursor muscle cells and the somatic component of the embryonic gonad ([31] and Fig S2). In *D14-3-3ε* mutant embryos, Zfh-1 levels were markedly reduced in all tissues where it is normally expressed and was completely absent from the mesodermal cells that constitute the somatic component of the gonad (Fig. 4B). The absence of Zfh-1 staining in the somatic component of the gonad was always coincident with reduction of pole cells in the gonads of *D14-3-3ε* mutant embryos. This result was confirmed in multiple experiments with representative results displayed in Fig. 4 B1-4. This reduction is especially apparent in Fig. 4B4 because of the better resolution afforded by detection of somatic cells in the far red which eliminates embryo auto-fluorescence. The differential reduction of Zfh-1 in the gonad is better illustrated in the hypomorphic *D14-3-3ε^J2B10^* homozygotes. Although Zfh-1 is reduced somewhat in mesodermal and nervous system tissues, the protein is totally absent in tissues surrounding the pole cells (Fig. 4B5 and 6). The fact that Zfh-1 continues to be expressed even at lower levels in other tissues implies either that it is not regulated by D14-3-3ε in these cells, or that Leo may be able to functionally compensate for its absence [13].

These results were confirmed independently by selective *D14-3-3ε* silencing with *UASIRepsilon* expression in tissues determined (Fig. 3B) to be required for normal pole cell migration and gonad formation. D14-3-3ε attenuation under Tub-Gal4 resulted in significant reduction in pole cell number and Zfh-1 levels in the presumptive somatic cells of the gonad, nearly to levels present in *D14-3-3ε^ex4^* homozygotes (Fig. 4C3 and 4C4). A more modest reduction in pole cells and Zfh-1 levels was observed under the posterior mesodermal driver NosVp16-Gal4 (Fig. 4C6). In contrast, no visible effects were precipitated under the broad mesodermal driver, How24B-Gal4 (Fig. 4C5), underscoring the cellular specificity of Zfh-1 loss upon D14-3-3ε attenuation. Collectively, these results strongly suggest that D14-3-3ε regulates the expression or stability of the Zfh-1 transcription factor, especially in the mesodermal component of the gonad. If this is the only role of D14-3-3ε in the somatic cells of the gonad, then by transgenically restoring the Zfh-1 in *D14-3-3ε* mutant embryos, we would expect the phenotype of the scattered pole cells to be reversed. Unfortunately, attempting to transgenically restore Zfh-1 in such homozygous mutant embryos with the Tub-Gal4 driver resulted in lethality, while the How24B-Gal4 and NosVp16-Gal4 were unable to rescue the pole cell deficit. Therefore, although in accord with our collective data, we were unable to further examine and confirm this hypothesis.

**Figure 5:**
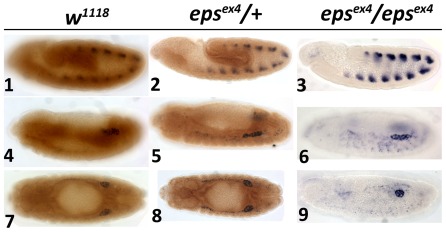
Mesodermally derived gonadal cells are present in *D14-3-3ε* mutant embryos. *In situ* hybridization for transcripts of the 412 retrotransposon as a marker of the mesodermal component of the gonad (blue) and immunohistochemical labeling of the D14-3-3ε protein (brown) shows that the somatic cells of the gonad are present in controls, heterozygous and homozygous D14-3-3ε mutant embryos as indicated at stage 9 (1, 4, 7), stage 11 (2, 5, 8) and stage 14 (3, 6, 9).

The absence of Zfh-1 from the presumptive mesodermally derived cells of the gonad may reflect their failure to differentiate as such, or survive altogether in *D14-3-3ε* mutant embryos. This was investigated using transcripts from the 412 retrotransposon which specifically mark the somatic cells of the gonad [22]. *In situ* hybridization experiments demonstrated that these gonadal cells survive and appeared of similar number in controls, heterozygous and homozygous *D14-3-3ε* mutant embryos (Fig. 5). Therefore, lack of D14-3-3ε only affects the expression or stability of Zfh-1 and not the viability of the somatic cells of the gonad. *In situ* hybridization experiments with a probe against *zfh-1* transcripts did not reveal differences between control and homozygous mutant embryos in the area typically occupied by the gonad, with the provision that the complicated and rather broad signal pattern may have obscured subtle differences (not shown). Therefore, we provisionally conclude that D14-3-3ε regulates Zfh-1 protein stability.

Collectively, our data demonstrate that D14-3-3ε is required within mesodermal cells which guide the pole cells during their migration and eventually participate in formation of the embryonic gonad by regulating Zfh-1 protein levels in the mesodermally derived somatic cells of the gonad.

## Discussion

Our results demonstrate that D14-3-3ε is necessary for migration of pole cells through the caudal mesoderm to the forming gonad. Although enriched within pole cells (Fig. 1B), D14-3-3ε is not required therein, but rather in SGPs and somatic gonadal cells, which mediate this migration and coalesce into the gonad. Therefore, within the pole cells D14-3-3ε likely serves a yet unknown function. Our data indicate that D14-3-3ε is not required for survival of somatic gonadal cells, but rather for their interaction with the pole cells, which in agreement with previous results [31] requires Zfh-1. An important experimental finding is that D14-3-3ε and Leo are not equivalent in mediating pole cell migration. This suggests specificity in D14-3-3ε-Zfh-1 interaction within somatic gonadal cells and perhaps in other tissues where the two are co-expressed.

14-3-3 proteins are known to bind transcription factors in a phosphorylation-dependent manner and modulate their subcellular localization and thus their activity [8]. For example, 14-3-3s bind and prevent entry into the nucleus of members of the Forkhead family of transcription factors [7]. Our results suggest that the highly consensus 14-3-3 binding site on Zfh-1, R-S-x-S-x-P, or the second more degenerate site at aas 824–837, are likely bound by D14-3-3ε. However there is no evidence currently that this occurs in the predicted phosphorylation-dependent manner. The prediction that Zfh-1 is phosphorylated at the second Serine of the consensus sequence, mediating D14-3-3ε binding will be addressed in future experiments.

Zfh-1 function is required for the development of the caudal visceral mesoderm, the SGPs and somatic gonadal cells [31]. Interestingly, D14-3-3ε loss seems to affect Zfh-1 levels primarily in the latter, since its pattern in most other tissues appears little affected (Fig. 4). It is possible that one or more Leo isoforms may functionally compensate for D14-3-3ε loss in all tissues other than SGPs and the somatic gonadal cells (Fig. 3), or that Zfh-1 activity in these cells is under unique regulation. Similarly, Leo isoforms were reported to compensate for D14-3-3ε loss in certain vital functions, but not in wing vein formation [13]. Alternatively, in comparison to other cell types, D14-3-3ε may serve a unique function with respect to Zfh-1 activity within SGPs and somatic gonadal cells. Therefore, although D14-3-3ε loss affects many tissues, the consequences are revealed in these cells because it regulates a critical factor for their development or survival.

We currently do not know whether D14-3-3ε is required for Zfh-1 transcription or translation *per se*. However, because its levels appear largely unaffected in other tissues of homozygous *D14-3-3ε* mutant embryos, we propose that D14-3-3ε is required for Zfh-1 entry into the nucleus and that the transcription factor is unstable in the cytoplasm in its absence. In fact, 14-3-3s are known to bind and prevent entry into the nucleus of members of the Forkhead family of transcription factors [7]. Destabilization of Zfh-1 upon D14-3-3ε loss may lead to fate changes or functional alterations because the transcription factor is required for expression of the nuclear protein Clift/Eyes absent. The Clift/Eyes absent protein is essential for SGPs and somatic gonadal cells differentiation [32] or functionality. The cells of the presumptive gonal do not appear to undergo a gross change in fate since they still express the 412 retrotransposon marker (Fig. 5). Rather, they apparently become unable to efficiently attract pole cells to coalesce into the gonad. Reduced attraction to the gonad likely results in pole cells not following the stereotypical migratory pathway, with a significant number of them remaining scattered in the caudal area of homozygous mutant embryos while few of them (∼7) reach the gonad. It appears the majority of these “lost” germ cells die around stages 10–12 (Fig. 1E). This is suggested by previous work indicating that primordial germ cell survival depends on their homing behavior and in ectopic cells die via apoptosis, probably initiated by a lack of localized survival factors [33].

Interestingly, we never observed complete loss of embryonic gonads in any of the D14-3-3ε null embryos even if they contained but a few pole cells. This is consistent with the evidence for two independent mechanisms that function in parallel and cooperatively for gonad formation. One is Zfh-1-dependent and the other requires Tin, a transcription factor without 14-3-3 binding sites (Table S2), which may mediate formation of the rudimentary gonads in the mutant embryos. Another potential 14-3-3-interacting protein which could participate in the process is AbdA, which contains 4 potential binding sites (Table S2) and is necessary for SGP specification [32], [34]. It is then possible that misregulation of this protein also contributes to the embryonic phenotype(s) upon D14-3-3ε loss. In addition, our *in silico* results suggest that the Trx protein with eight 14-3-3 binding sites of three different consensus variants, is likely to be a *bona fide* 14-3-3 interacting protein, which has also been proposed to participate in germ cell migration and embryonic gonad formation [22].

Oocytes and embryos lacking D14-3-3ε display gross defects in anterior–posterior polarization, rendering them unviable and offers an explanation for the inability to propagate the viable homozygotes [35]. In contrast, homozygous embryos from heterozygous mothers such as the ones we focused on in this work, although they hatch with 25–30% of normal pole cells as we have shown, they nevertheless do have a germ line. Provided that these remaining pole cells become germ line stem cells [36], this does not account for the small number of eggs laid by homozygous females (Fig. 1A) and the inability of such males to fertilize control females (Table 1). In fact, homozygous females contain very few eggs in rudimentary germaria (Fig S3), suggesting that D14-3-3ε may also play a role either in the transition of pole cells to stem cells, or maintenance of their stem cell-state. Ongoing preliminary experiments suggest the latter and will also be the focus of future investigations.

Seen in a wider context, this study focused on mechanisms of directed cellular migration. In addition to that of pole cells, such mechanisms underlie other morphogenetic movements in the embryo that give rise to musculature and nervous system, but also exhibit common features with the migration of metastatic cancer cells [37]. Therefore, 14-3-3ε or other members of the protein family may be involved such processes in vertebrates. In fact, similar to Drosophila, chemotaxis is vital for primordial germ cell development in zebrafish and mouse, as Sdf-1/CXCR4 directs their migration and significantly the same chemokine receptor may be involved in metastasis of many tumor cell-types [38].

## Materials and Methods

### Drosophila culture and strains

Drosophila were cultured in standard cornmeal, soy flour and sugar food supplemented with CaCl_2_ at 21–23°C [13]. The mutant alleles *D14-3-3ε^l(3)j2B10^* and *D14-3-3ε^ex4^* and the revertant *D14-3-3ε^ex5^* were described previously and their genetic background was normalized to our resident Cantonized *w^1118^*. Homozygous embryos were identified from heterozygotes with the aid of the fluorescent balancer chromosome TM3GFP, (TM3Ser^1^, P{GAL4-Hsp70.PB}TR2, P{UAS-GFP.Y}TR2), expressing ubiquitously the Green Fluorescent Protein [39]. The following GAL4 drivers were obtained from the Bloomington Stock Centre: P{tubP-GAL4}LL7 (abbreviated in the text and figures as Tub-Gal4 [ubiquitousexpression]), P{Act5C-GAL4}25FO1 (abbreviated as Act-Gal4 [broad expression]), P{GAL4::VP16-nos.UTR}MVD2 (abbreviated as NosVp16-Gal4 [expression in embryonic posterior mesoderm and pole cells]) and P{GawB}how[24B] (abbreviated as How24B-Gal4 [expression in mesodermal cells]), and the bovine Tau-expressing UAS-*b-Tau* transgene. We generated and used the following transgenic strains: UAS-*D14-3-3ε^Η^*, UAS-*D14-3-3ε^L^*, UAS-*leoI* and UAS-*leoII* and the RNA-interference (RNAi)-mediating UAS-*IRD14-3-3ε*. This transgene expresses an inverted repeat RNA from the *D14-3-3ε* gene. To generate UAS-*IRD14-3-3ε*, a 990 bp XbaI fragment of the gene containing the complete cDNA, was subcloned into pUAST [40] in the same orientation as that of the UAS controlled transcription. Subsequently, another 400 bp fragment, containing the gene 5′ end to the unique EcoRI site, was subcloned into the same pUAST plasmid in the opposite orientation (Fig. S1) and transformants were obtained in the *w^1118^* background using standard methods. Of the multiple transformants obtained, the line bearing an insert in the X chromosome was the most effective in silencing the endogenous gene (Fig. S1) and was used for most experiments shown in the figures.

### Fertility and pole cell measurements

For single fly experiments mutant females were observed to mate with *w^1118^* males, or conversely mutant males were observed to mate with control females in food vials and remained there undisturbed for 7 days. They were then moved to new vials for another 7 days. If even a single larva or adult emerged from any of the two vials per mating the cross was scored as fertile. For multiple fly crosses, 20–30 females were placed with males in a vial for 16 hours at 21–23°C. Thereafter, they were transferred in an egg collection cage made from a polyethylene tri-pour beakers and embryos were collected on agar and apple juice plates. Counts continued for 3 days for every cross and were carried out by means of a Zeiss Stemi V2 stereoscope. The results were analyzed statistically with the SAS JMP software as suggested by Rolf and Sokal.

### Immunohistochemistry

Embryos were collected on agar-apple juice plates, dechorionated and fixed in 43.2 mM Hepes, 0.96 mM MgSO_4_, 0.48 mM EGTA, pH 6.9, 1.6% formaldehyde in 59% heptane. Subsequently, they were rinsed with methanol, 5% EGTA. The embryos were hydrated in BBT (140 mM NaCl, 2.7 mM KCl, 4.3 mM Na_2_HPO_4_, 1.4 mM KH_2_PO_4_, pH 7.3, 0.1% Tween-20, 1%, BSA) and “blocked” for 1 hour in BBT-250 (BBT, 250 mM NaCl), 10% normal goat serum (NGS). Subsequently, they were incubated with primary antibodies in 5% NGS BBT-250 as follows: chicken anti-D14-3-3ε 1∶3000, rabbit anti-vasa 1∶3000 (P. Lasko), mouse anti-GFP 1∶5000 (Molecular Probes), mouse anti-Tau 1∶3000 (Developmental Hybridoma Studies Bank, University of Iowa City, IA), and mouse anti-Zfh-1 (Z. C. Lai). Fluorescent secondary antibodies conjugated to Alexa-555, Alexa-488 and Alexa-647 (Molecular Probes, Eugene, OR) were used at 1∶2000. Images from 2–3 µm optical sections were captured on a Biorad 2100 confocal microscope. The homozygous embryos were indentified based on lack of signal from the GFP-bearing balancer chromosome. Balancer chromosome homozygotes were excluded from analyses due to their abnormal appearance.

Pole cell counts were carried out using a Biorad 2100 confocal microscope and the numbers refer to pole cell number per gonad. The results were analyzed statistically as detailed above [41].

### In situ hybridization

Embryos were collected on agar and apple juice plates, dechorionated and fixed in 43.2 mM HEPES, 0.96 mM MgSO_4_, 0.48 mM EGTA, pH 6.9, 1.6% formaldehyde in 59% heptane. Subsequently, they were rinsed with 50% methanol and 50% PBT (140 mM NaCl, 2.7 mM KCl, 4.3 mM Na_2_HPO_4_, 1.4 mM KH_2_PO_4_, pH 7.3, 0.1% Tween-20). The embryos were hydrated in PBT and incubated for 3 minutes in 40 µg/ml proteinase K, rinsed in PBT and fixed again for 25 minutes in PBT and 5% formaldehyde. They were then transferred in hybridization solution for 1–2 hours at 55°C. Finally, 40 µl of single-stranded RNA probe in complete hybridization solution was added for at least 12 hours at 55°C. The 412 retrotransposon gene RNA probe was prepared and used as previously described [31]. After hybridization and washes, the embryos were stained with the chicken anti-D14-3-3ε antibody (1∶3000) and with anti-chicken biotin a 1∶2000, as described previously [42].

### Western blot analysis

Staged embryos or entire 2–3 day old flies as indicated were homogenized in 1× Laemli solution (50 mM Tris pH 6.8, 100 mM DTT, 5% 2-mercaptoethanol, 2% SDS, 10% glycerol, and 0,01% bromophenol blue). The protein extracts were heated for 10 minutes at 95°C and the denatured proteins were separated by SDS-PAGE electrophoresis. The electrophoretically separated proteins were transferred onto PVDF membranes and the D14-3-3ε and β-Tubulin (Developmental Hybridoma Studies Bank, University of Iowa City, IA) proteins were detected with the respective antibodies (at 1∶2000 and 1∶500). The secondary anti-chicken and anti-mouse antibodies were used at 1∶2000 and 1∶4000 respectively. The proteins were detected by chemiluminescence (PIERCE).

## Supporting Information

Figure S1 A. A map representing the salient features if the “head to head” construction utilized to generate the UAS*IRepsilon* RNAi-mediating transgene in the pUAST vector. B. D14-3-3ε abrogation by two independent transgene insetions visualized in Western blots of late embryo extracts. The two transgenic lines shown here were utilized for all experiments, with all data shown in the figures obtained with the insertion on the X chromosome (UAS*IRepsilon* ^X^), but verified independently with the other on the second (UAS*IRepsilon* ^2^).
(JPG)

Figure S2 **The distribution of Zfh-1 protein (green) in wild type embryos stages 8 (1), 11 (2) and 14 (3), shown with reference to the nervous system labeled with the 22c10 monoclonal antibody (blue) and the pole cells labeled with anti-Vasa (red).** Zfh-1-containing cells of the gonad are indicated by arrows, whereas arrowheads point to cells expressing high levels of the protein either in the nervous system or the mesoderm.
(TIF)

Figure S3 **Saggital sections of adult female abdomens of the indicated genotypes stained with anti-D14-3-3ε.** The arrow indicates the single apparent oocyte present in the abdomen of the mutant homozygous female. Entire flies were fixed in Carnoy's fixative, paraffinized, sectioned and processed for immunohistochemistry as previously described [43], [44].
(TIF)

Table S1 **Rescue of the fertility deficit of** ***D14-3-3ε^ex4^*** **(2) and** ***D14-3-3ε^J2B10^*** **homozygotes with conditional expression of heat-shock inducible full length transgenes yielding high (** ***hsD14-3-3ε^H^*** **) and low (** ***hsD14-3-3ε*** **^L^) levels of the protein.** Experimental conditions are as detailed on the table and the number of single crosses that yielded larvae (% Fertile) over the total number of animals crossed (# crossed) per genotype is reported.
(DOC)

Table S2 **Results from the** ***in silico*** **search for 14-3-3 binding sites on proteins known to involved in pole cell migration and embryonic gonad formation** [**22**] **.** The number (# hits) of sequences on each target protein matching the indicated 14-3-3 binding motif is shown, as well as the exact sequence and its location in the protein (sequences).
(DOC)
