# Supplementary figures and images for: 14-3-3ε Is Required for Germ Cell Migration in Drosophila
Source: PLoS One. 2012 May 30;7(5):e36702. doi: 10.1371/journal.pone.0036702 (PMC3364263; doi:10.1371/journal.pone.0036702)

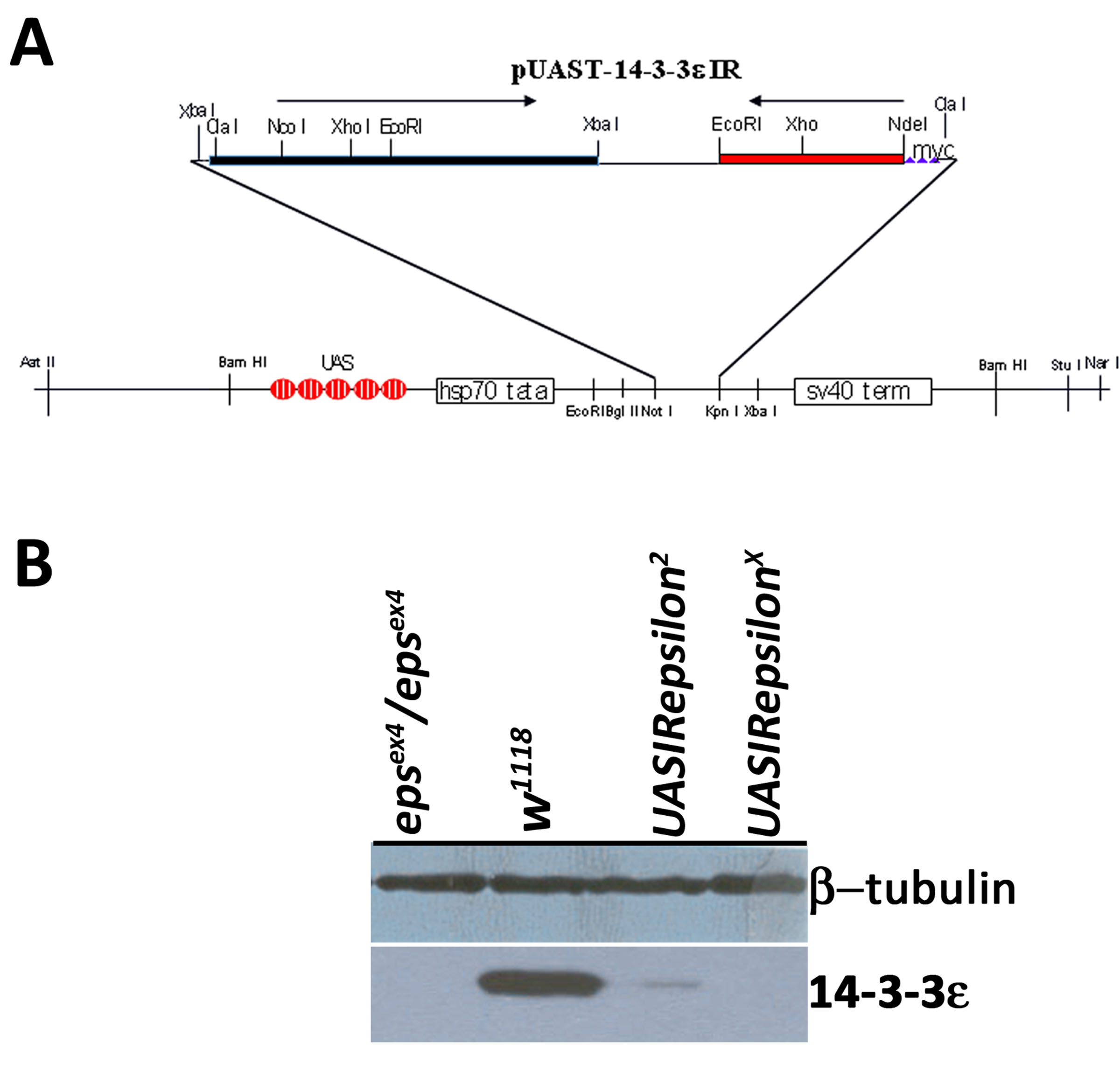

Supplement: Figure S1 — A. A map representing the salient features if the “head to head” construction utilized to generate the UASIRepsilon RNAi-mediating transgene in the pUAST vector. B. D14-3-3ε abrogation by two independent transgene insetions visualized in Western blots of late embryo extracts. The two transgenic lines shown here were utilized for all experiments, with all data shown in the figures obtained with the insertion on the X chromosome (UASIRepsilon X), but verified independently with the other on the second (UASIRepsilon 2). (JPG) [file pone.0036702.s001.jpg]

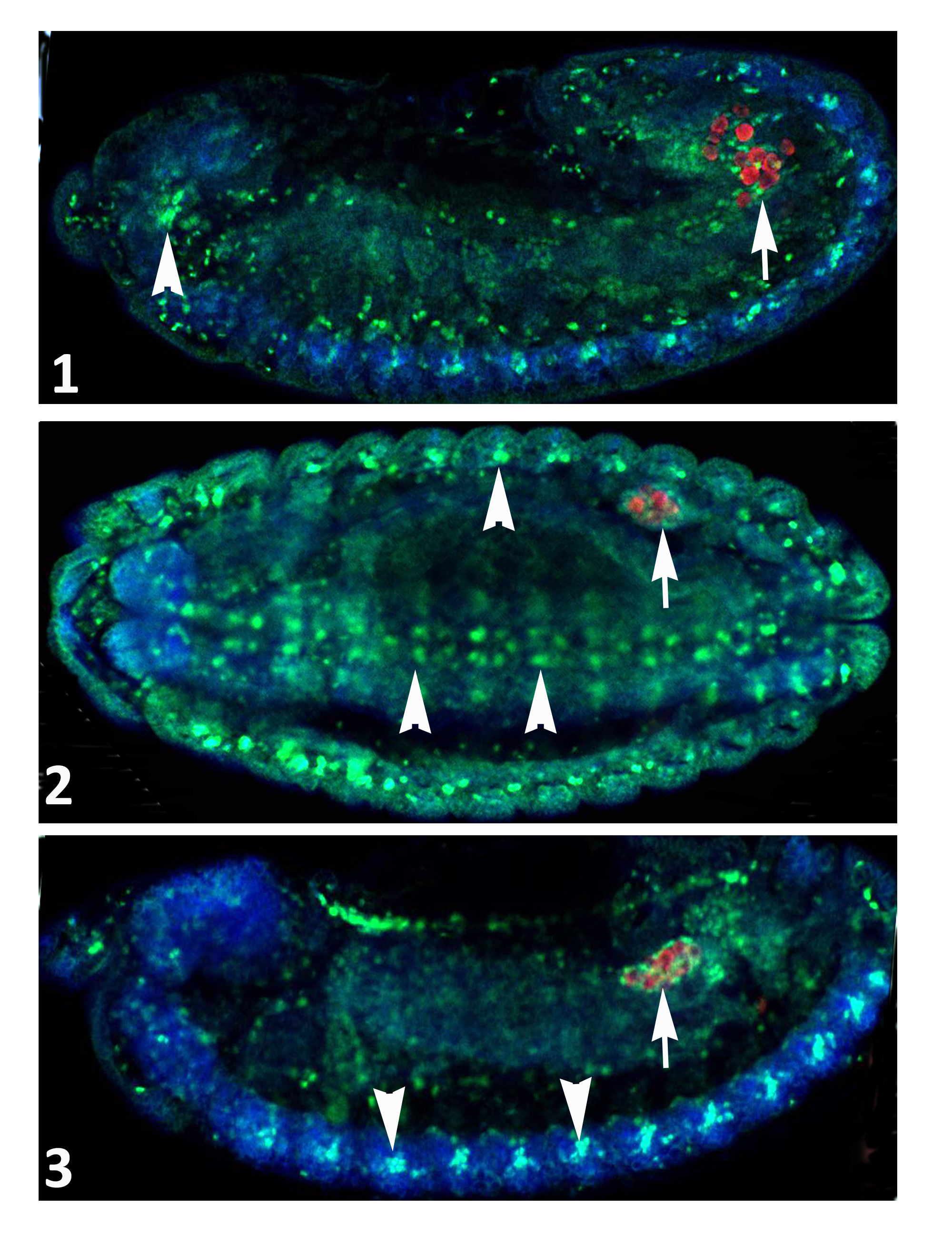

Supplement: Figure S2 — The distribution of Zfh-1 protein (green) in wild type embryos stages 8 (1), 11 (2) and 14 (3), shown with reference to the nervous system labeled with the 22c10 monoclonal antibody (blue) and the pole cells labeled with anti-Vasa (red). Zfh-1-containing cells of the gonad are indicated by arrows, whereas arrowheads point to cells expressing high levels of the protein either in the nervous system or the mesoderm. (TIF) [file pone.0036702.s002.tif]

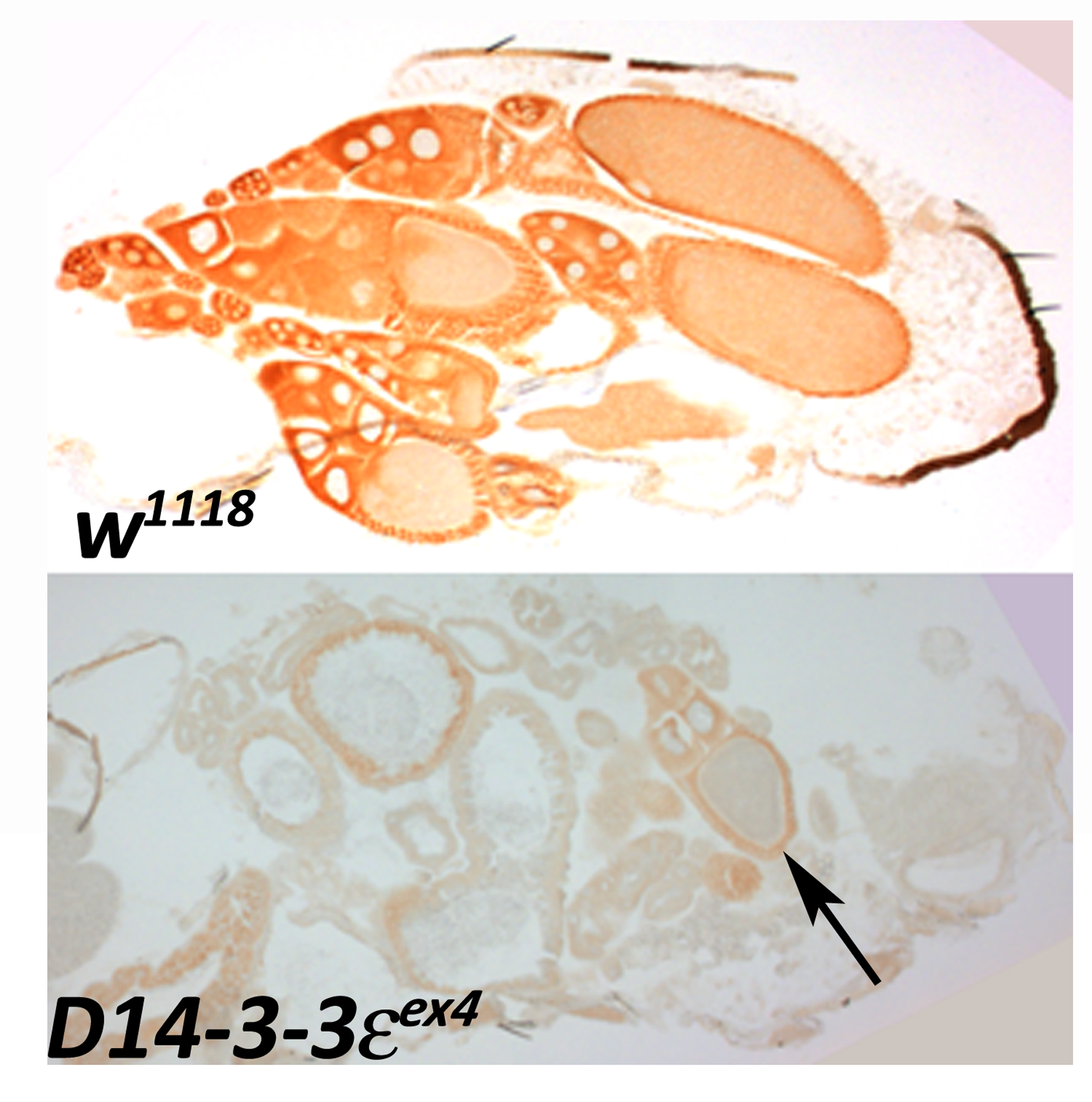

Supplement: Figure S3 — Saggital sections of adult female abdomens of the indicated genotypes stained with anti-D14-3-3ε. The arrow indicates the single apparent oocyte present in the abdomen of the mutant homozygous female. Entire flies were fixed in Carnoy's fixative, paraffinized, sectioned and processed for immunohistochemistry as previously described [43], [44]. (TIF) [file pone.0036702.s003.tif]
