# Supplementary material for: 14-3-3ε Is Required for Germ Cell Migration in Drosophila
Source: PLoS One. 2012 May 30;7(5):e36702. doi: 10.1371/journal.pone.0036702 (PMC3364263; doi:10.1371/journal.pone.0036702)
Supplement: Table S1 — Rescue of the fertility deficit of D14-3-3εex4 (2) and D14-3-3εJ2B10 homozygotes with conditional expression of heat-shock inducible full length transgenes yielding high ( hsD14-3-3εH ) and low ( hsD14-3-3ε L) levels of the protein. Experimental conditions are as detailed on the table and the number of single crosses that yielded larvae (% Fertile) over the total number of animals crossed (# crossed) per genotype is reported. (DOC) [file pone.0036702.s004.doc]

# Supplemental Table 1

# Rescue of sterility of *D14-3-3ε*mutants

| **14-3-3**  **Allele** | **transgene** | **Female**  **# Crossed % Fertile** ^a^ | | **Male**  **# Crossed % Fertile** ^a^ | |
| --- | --- | --- | --- | --- | --- |
| *D14-3-3ε^l(3)j2B10^* | --- | 30 | 0 | 25 | 0 |
| *D14-3-3ε^l(3)j2B10^* | *hsD14-3-3ε^L^* (18) | 22 | 0 | 24 | 0 |
| *D14-3-3ε^l(3)j2B10^* | *hsD14-3-3ε^L^* (RT) | 27 | 0 | 24 | 0 |
| *D14-3-3ε^l(3)j2B10^* | *hsD14-3-3ε^L^* (HS) | 24 | 100 | 26 | 100 |
| *D14-3-3ε^l(3)j2B10^* | *hsD14-3-3ε^H^* (18) | 25 | 0 | 23 | 0 |
| *D14-3-3ε^l(3)j2B10^* | *hsD14-3-3ε^H^* (RT) | 25 | 20 | 24 | 25 |
| *D14-3-3ε^l(3)j2B10^* | *hsD14-3-3ε^H^* (HS) | 25 | 100 | 25 | 100 |
| *D14-3-3ε^ex4^* | --- | 25 | 0 | 25 | 0 |
| *D14-3-3ε^ex4^* | *hsD14-3-3ε^L^* (18) | 23 | 0 | 25 | 0 |
| *D14-3-3ε^ex4^* | *hsD14-3-3ε^L^* (RT) | 25 | 0 | 25 | 0 |
| *D14-3-3ε^ex4^* | *hsD14-3-3ε^L^* (HS) | 28 | 66 | 22 | 50 |
| *D14-3-3ε^ex4^* | *hsD14-3-3ε^H^* (18) | 25 | 0 | 24 | 0 |
| *D14-3-3ε^ex4^* | *hsD14-3-3ε^H^* (RT) | 25 | 8 | 27 | 7 |
| *D14-3-3ε^ex4^* | *hsD14-3-3ε^H^* (HS) | 24 | 100 | 25 | 100 |
|  |  |  |  |  |  |

^a^ The number of single crosses that yielded larvae over the total number of animals crossed per genotype.

HS: Three daily 20-minute heat shocks at 32°C through development to adulthood.

RT: Constant 20-22°C. 18: Constant 18°C.
