# Supplementary material for: 14-3-3ε Is Required for Germ Cell Migration in Drosophila
Source: PLoS One. 2012 May 30;7(5):e36702. doi: 10.1371/journal.pone.0036702 (PMC3364263; doi:10.1371/journal.pone.0036702)
Supplement: Table S2 — Results from the in silico search for 14-3-3 binding sites on proteins known to involved in pole cell migration and embryonic gonad formation [22] . The number (# hits) of sequences on each target protein matching the indicated 14-3-3 binding motif is shown, as well as the exact sequence and its location in the protein (sequences). (DOC) [file pone.0036702.s005.doc]

| **PROTEIN** | **#HITS** | **PATTERN** | **SEQUENCES** |
| --- | --- | --- | --- |
| Abd B  Foi  Htl  Pgc  Tin  Tre  Tre-1  Wun  Wun2 | 0  0  0  0  0  0  0  0  0 |  |  |
| Abd A | 4 | X_(3)_-S-X-S-X_(3)_-S-X_(2)_-S-X | - 1. aaaSaSasvSasSs   2. asaSaSvsaSssSn   3. sscSpSpsaSgsSs   4. gsaSaSasaSaaSs |
| Clb/Hmgcr | 1 | X_(3)_-S-X-S-X_(3)_-S-X_(2)_-S-X | 56-69 aggSgSgagSgaSg |
| Hkb | 2 | X_(3)_-S-X-S-X_(3)_-S-X_(2)_-S-X | - 1. dfsSaSssaSssSn   2. ssaSsSassSsnSs |
| Srp | 1 | X_(3)_-S-X-S-X_(3)_-S-X_(2)_-S-X | - 1. smgStSlspSamSh |
| Trx | 8 | X_(3)_-S-X-S-X_(3)_-S-X_(2)_-S-X  R-X_(3)_- S-X-P  Y-T-V | - 1. gssSgSstgSgsSg   2. fsaStSvtsSgrSs   511-524 assSsSnqeSgsSs  658-671 fgtSsSsagStaSt  3031-3044 fstSsSsssSncSl  181-187 RssgSsP  1934-1936 YTV  1956-1958 YTV |
| Zfh-1 | 2 | R-S-X-S-X-P  X_(3)_-S-X-S-X_(3)_-S-X_(2)_-S-X | - 1. RStSsP   2. rkfStSasmSpaSi |

**Supplemental Table 2.**
